# MicroRNAs Responding to Space Radiation

**DOI:** 10.3390/ijms21186603

**Published:** 2020-09-09

**Authors:** Yujie Yan, Kunlan Zhang, Guangming Zhou, Wentao Hu

**Affiliations:** State Key Laboratory of Radiation Medicine and Protection, School of Radiation Medicine and Protection, Collaborative Innovation Center of Radiological Medicine of Jiangsu Higher Education Institutions, Soochow University, Suzhou 215123, China; yjyan@stu.suda.edu.cn (Y.Y.); 1630509008@stu.suda.edu.cn (K.Z.)

**Keywords:** microRNA, space radiation, risk assessment

## Abstract

High-energy and high-atom-number (HZE) space radiation poses an inevitable potential threat to astronauts on deep space exploration missions. Compared with low-LET radiation, high-energy and high-LET radiation in space is more efficient in inducing clustered DNA damage with more serious biological consequences, such as carcinogenesis, central nervous system injury and degenerative disease. Space radiation also causes epigenetic changes in addition to inducing damage at the DNA level. Considering the important roles of microRNAs in the regulation of biological responses of radiation, we systematically reviewed both expression profiling and functional studies relating to microRNAs responding to space radiation as well as to space compound environment. Finally, the directions for improvement of the research related to microRNAs responding to space radiation are proposed. A better understanding of the functions and underlying mechanisms of the microRNAs responding to space radiation is of significance to both space radiation risk assessment and therapy development for lesions caused by space radiation.

## 1. MicroRNAs

MicroRNAs (miRNAs) are a class of small single-stranded endogenous noncoding RNAs of 18-24 nucleotides at length, which are identified in various organisms, including mammals, plants, and many microorganisms. They are transcribed from the genomes by RNA polymerase II or RNA polymerase III, capable of suppressing the expression of a large number of genes by binding to the 3′-untranslated region (3′-UTR) of their transcripts [1].

Early studies have found that most of miRNA genes are located in the intergenic region 1 kb away from the annotated genes, while some of miRNA genes are located in the introns of known genes and share the transcriptional elements with their host genes. It is reported that about 50% of miRNA gene is located closely to other miRNAs as a cluster [2,3]. Most miRNA genes have their own promoters and the clustered miRNAs come from the polycistronic transcripts [4,5]. Most of miRNAs are transcribed from the genomes by RNA polymerase II or RNA polymerase III under strict regulation [4]. It was found by Bradley that 70% of the mammalian miRNA genes were located in a specific transcription unit, and 117 of 232 miRNAs were located in the introns, of which 90 were located in the introns of protein-encoding genes, and 27 were located in non-coding RNAs in the introns. Thus, miRNAs can be divided into three types according to their location: the exonic miRNAs located in the non-coding transcription units, the intronic miRNAs located in the non-coding transcription units, and the intronic miRNAs in the protein encoding units [6].

The primary transcription product of a miRNA gene is primary miRNA (pri-miRNA), which is thousands of nucleotides long and contains a hairpin structure. Once the primary miRNA is produced, its 5′ end is added with a methylation hat while the 3′ end is polyadenylated [7]. The modified primary miRNA is first cut by the nuclear ribonuclease III (RNase III) Drosha in the nucleus to produce about 70 nucleotides long precursor miRNA molecules (precursor miRNA, pre-miRNA) [8]. It is generally believed that the residual flanking sequence will be degraded in the nucleus, but its specific function remains to be clarified. Drosha is a conserved protein of 160 kDa size containing two series of RNase III domains (RIIIDs) and a double stranded RNA binding domain (dsRBD) [9], which in the human body associates with DiGeorge syndrome critical region gene 8 (DGCR8) to form a microprocessor complex of 650 kDa [10]. At present, it is believed that DGCR8 can assist Drosha to identify the substrate, and the determinant of the specificity for substrate recognition is the structure of pri-miRNA [11,12,13].

**Figure 1:**
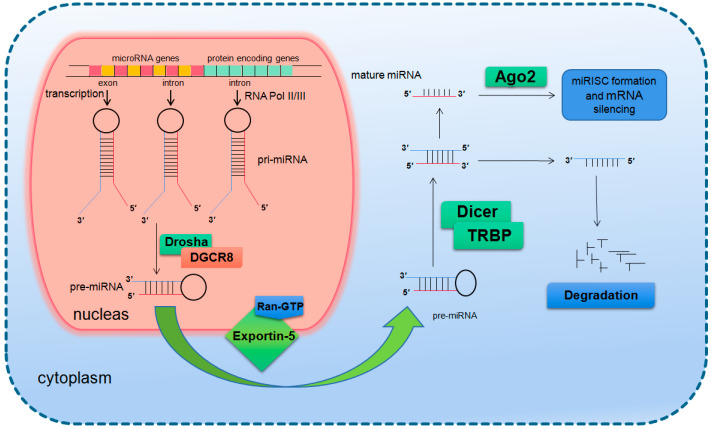
Basic miRNA processing pathway.

In mammals, the two-step splicing of pri-miRNA occurs in the nucleus and cytoplasm (Figure 1). The nuclear export of a pre-miRNA is mediated by nuclear transport receptor exportin-5. It combines pre-miRNA and co-factor Ran-GTP in the nucleus. Once exported, GTP is hydrolyzed to GDP, resulting in the release of pre-miRNA from the transport complex. When exportin-5 is knocked-down, the amounts of both pre-miRNA and mature miRNA in the cytoplasm decrease, but the pre-miRNA in the nucleus does not accumulate, which indicates that pre-miRNA is extremely unstable in the nucleus or gets stabilized by binding with exportin-5 [14,15]. The homologues of Drosha and DGCR8/Pasha were not found in plants and yeast. The study in Arabidopsis found the nucleoprotein Dcl1 was one of the Dicer-like proteins and was involved in processing miRNA [16,17].

After being exported to the cytoplasm, the pre-miRNA is further spliced by RNase III nuclease Dicer to generate ~22 nt double stranded RNA molecules. It is reported that the knockout of Dicer in *Caenorhabditis elegans* (*C. elegans*) causes the accumulation of pre-miRNA and the decrease of mature miRNA [18,19], providing direct evidence for Dicer as a RNA enzyme in the maturation process of miRNA. Dicer is about 200 kDa of the multi-domain protein, which contains two RIIIDs domains and a dsRBD domain like Drosha, and the difference is that Dicer contains a long N short sequence, including a DEAD-BOX RNA helicase domain, a DUF283 domain and a PAZ domain. The PAZ domain is also found in the Argonaute family of proteins, which specifically binds to the 3′ terminal of small RNA molecules [20,21]. There is only one kind of Dicer in mammals and nematodes, which plays a key role in the biogenesis of siRNA and miRNA [22,23]. The miRNA dimer is subsequently integrated into the complex called miRNP (ribonucleoprotein complex containing miRNA) or miRISC (miRNA-induced silencing complex). The life span of miRNA dimer is short. Once combined with Ago protein, the double strands are quickly unraveled, a chain is stabilized and the other is lost. The retention or degradation of the chain depends on its thermodynamic stability [24,25,26]. The mammalian Dicer/Ago/miRNA complex also interacts with a number of other proteins, such as Gemin3, Gemin4, Mov10, and GW182 [27,28,29,30].

Since the first miRNA Lin-4 was identified in nematode in 1993 as a development regulation gene, the number of miRNAs found in all species has reached more than 38,000 and the number of human miRNAs also exceeded 2600 (http://www.mirbase.org/). It is presumed that more than 60% of the human genes are regulated by miRNAs [31,32]. Precious studies suggest that miRNAs regulate gene expression through translational inhibition and/or degradation of target mRNAs [33,34]. There is no final conclusion on which mechanism will play a major role. It is speculated that the number, type and location of mismatch in miRNA/mRNA dimers as well as the cell type determines the triggering of degradation or translation inhibition [35,36]. A miRNA interacts with its target genes to form a regulatory network, which acts in almost all life processes. It has been shown by many studies that miRNAs are a key group of important modulators in biological processes and play important roles in development [37,38], organogenesis [39,40], cell proliferation and differentiation [41], cell cycle regulation [41,42], cell apoptosis [43,44,45], aging [46,47,48], pathogenesis [49,50], cellular response to stresses [51,52,53,54], etc.

As a key modulator in radiation response of human and other organisms, miRNAs have been found to respond to various radiation (both ionizing radiation (IR) and non-ionizing radiation) as well as to regulate a lot of genes involved in the cellular radiation response [55,56,57]. As a key radiation type confronted by the astronauts in the manned spaceflight missions, high-energy and high-Z (HZE) particles-related biological effects and the underlying mechanisms have attracted much attention from space radiobiologists. In particular research on HZE particle radiation-induced miRNAs and their roles in the regulation of the corresponding biological effects has made great progress.

## 2. Space Radiation and the Health Effects

With the deepening of space exploration, the duration of astronauts’ permanence in space will become longer and longer. The safety evaluation of space explorations beyond the low Earth orbit (LEO) inevitably is one of the most important topics in space science since the radiation levels exceed those routinely received by terrestrial radiation workers, or astronauts in near-Earth orbits such as the International Space Station (ISS). Because of the lack of atmosphere and magnetic field and thereby important shielding properties the only shielding measure is the spacecraft bulkhead and high energy space radiation is an unavoidable danger for astronauts in manned space missions.

Basically, there are three main sources of space radiation: (1) Trapped low Earth orbit (LEO) radiation, in which the main components are protons and electrons with relatively low energy. (2) Incidental and high dose radiation from solar particle events (SPEs). Solar flares emit a large number of high-energy protons, and a small amount of α particles and heavy ions. (3) Low dose rate galactic cosmic rays (GCR). The protons account for about 85%, followed by 14% α particles and 1% of heavy ions. Although low in dose and dose rate, GCR is highly energetic and powerfully penetrating. There are no shielding materials capable of blocking it completely. Exposure of astronauts to GCR causes clustered DNA damages that are difficult to repair, which are strongly lethal, mutagenic and carcinogenic [58,59,60]. Heavy ions are high in ionization density (linear energy transfer, LET), although their abundance is low [61,62,63]. In addition to the abovementioned radiation, secondary particles produced by these primary particles through the shielding materials form a secondary radiation environment, including photons, electrons, protons and neutrons, which can also lead to biological effects such as DNA damage, gene mutation, cell transformation, and other biological effects [64].

The human experience in space flights is only about six decades old, inaugurated by the first complete Earth orbit flight by Yuri Gagarin, and currently continuing on the International Space Station. To date, manned missions have been limited to near-Earth orbits, with the moon as our farthest destination from Earth. Historical space radiation career exposures for astronauts from all NASA missions through December 1999 (including early Mercury, Gemini, STS, and Apollo Missions) involved total exposures of less than about 20 mSv [65]. With the advent of Skylab and MIR, total career exposure levels increased to a maximum of nearly 200 mSv. It is estimated that astronauts will be exposed to 1.84 ± 0.30 mSv/day of GCR in interplanetary space and 0.64 ± 0.12 mSv/day of GCR on the Mars surface, amounting to a total mission dose equivalent of ~1.01 Sv for a round trip to Mars with 180 days (each way) cruise, and 500 days stay on the Mars surface for a particular solar cycle [66]. Besides, radiation tissue equivalent doses deposited in blood-forming organs when encountering a large SPE may reach 1.93 Sv even behind 5 g/cm^2^ Al shielding [67]. Missions into deep space, due to the requisite longer duration of the planned missions, may pose greater risks due to the increased potential for exposure to complex radiation fields comprised of a broad range of radiation types and energies from cosmic and unpredictable solar sources. Ionizing radiation prevalent in space such as protons, carbon, argon and iron ions covers both a broad range of energies and a highly diverse range of radiation quality. LETs of these particles range from <10 to >200 keV/µm. Protons are more prevalent with relatively low LET values, whereas the iron ions are relatively rare, but with high LET values. Dose rate measured in LEO is of the order of 1 mSv/day, but will be higher on missions to Mars as mentioned above. It is an enormous and complex task to assess the biological and clinical effects of all possible space radiation scenarios. Reminders of the presence of low-flux particle radiation fields have graphically been visualized in light-flash phenomena experienced by many space travelers [68]. Evidence exists from the accelerator-based human exposures with muons [69], pions [70], helium ions [71], carbon ions [72], and nitrogen ions [73]. Visual phenomena have also been noted by human subjects on exposure to neutrons of various energies. Several human subjects saw a multitude of bright colorless flashes on exposure to neutrons, which were described as “a bunch of stars moving or blinking” and are similar to light flashes and streaks seen by astronauts on translunar flight. These phenomena are caused by interaction with retinal rods by proton recoils and by α particles released from neutron reactions with carbon and oxygen [74,75,76]. However, space radiation exposure influences multiple organs and physiological systems in complicated ways. NASA has classified the biomedical consequences into four risk areas [77]: (1) Degenerative tissue effects from radiation exposure, e.g., cardiovascular disease, cataract formation, and premature aging; (2) Radiation-induced carcinogenesis; (3) CNS (central nervous system) injury caused by radiation exposure, leading to deficits in cognitive and executive function, inducing fatigue, and degrading crew performance; (4) Radiation syndromes caused by SPE. The high doses of radiations from large SPEs induce acute radiation syndrome effects, such as nausea, emesis, haemorrhaging, or, possibly, even death. However, the underlying molecular mechanisms are still under considerations.

## 3. MiRNAs Involved in the Biological Responses to Space Radiation

Up to now, a number of studies have found that the biological responses to space radiation are related to miRNAs, as demonstrated in Table 1. Khan et al. examined the miRNA expression in selected mouse organs (testis, brain, and liver) exposed to whole-body proton irradiation (2 Gy). By bioinformatics analysis they revealed dysregulation of 14 miRNAs in mouse testis, 8 in liver, and 8 in brain and a possible mechanism of proton particle involvement in the onset of tumorigenesis [78]. Templin et al. studied the expression changes of miRNA derived from mouse blood using quantitative real-time polymerase chain reaction (qRT-PCR) in mice exposed to 600 MeV protons at doses of 0.5 or 1.0 Gy. They found 26 miRNAs were differentially expressed and mouse blood miRNA signatures are radiation type- and dose-dependent [79].

Besides the involvement of miRNAs in response to proton radiation, they were also found to participate in the biological responses to particles of higher LET, such as α particle and heavy ions. Kovalchuk et al. analyzed microRNAome changes in bystander tissues after 5.4 Gy α-particle microbeam irradiation of 3-D artificial human tissues using miRNA microarrays and found that miRNAs play a profound role in the manifestation of late radiation-induced bystander effect (RIBE) end points [80]. Chauhan et al. studied the miRNA expression patterns in three human cell lines (A549, THP-1 and HFL) exposed to 0.5 Gy, 1.0 Gy and 1.5 Gy of α-particles at a low dose-rate (0.98 ± 0.01 Gy/h), and found cell-specific responses of 13 miRNAs. Besides, bioinformatics analysis suggested the α-particle induced miRNA mapped to target genes related to ribosomal assembly, lung carcinoma development, TGF-β signaling, cell communication and keratin sulfate [81]. Nie et al. studied the malignant transformation of immortalized human bronchial epithelial cells (BEAS-2B) irradiated by 0.25 Gy α-particles using miRNA-mRNA networks. Sixty-eight miRNAs were found to be dysregulated, among which miR-107 and miR-494 were predicted to play a role in α-particles-mediated cellular malignant transformation processes by bioinformatics analysis [82].

Templin et al. also compared the miRNA expression signatures in peripheral blood of mice exposed to either γ-rays or ^56^Fe ions and found that miRNA expression signatures were radiation type-specific and dose- and time-dependent [83]. He et al. investigated the toxicity in testis of mice following enterocoelia irradiation with 2 Gy carbon ions by miRNA sequencing and bioinformatics analyses. Differentially expressed miRNAs were found to be involved in the regulation of metabolism, development, and reproduction [84]. Wei et al. analyzed miRNA expression profiles with miRNA PCR arrays at 24 h post heavy ion irradiation, and developed a universal model to predict the degree of exposure to different radiation types with high sensitivity and specificity based on five miRNAs (miR-183-5p, miR-9-3p, miR-200b-5p, miR-342-3p and miR-574-5p) that showed a significant response to 0.1–2 Gy of carbon-ion, iron-ion or X-rays [85]. In another study, this team exposed Kunming mice to different doses of carbon ions and X-rays and found two miRNAs (let-7a-5p, miR-200b-5p) in the serum of irradiated mice were up-regulated significantly and exhibited dose- (0.1~2 Gy) and time-dependence (6~72 h), which may serve as potential noninvasive indicators for space radiation [86].

In addition to the characteristic of being rich in high LET radiation, the effects of space radiation on astronauts are affected by a variety of other space environmental factors, such as microgravity, weak magnetic field, and short circadian rhythm. As shown in Table 2, several studies have been carried out to identify the miRNAs participating in the regulation of compound effect of space radiation and other space environment factors. Researchers from Padua University analyzed miRNA expression profile in human peripheral blood lymphocytes (PBL) incubated for 4 and 24 h in normal gravity (1 g) and in modeled microgravity (MMG) during the repair time after irradiation with 0.2 or 2 Gy of γ-rays. They found that MMG altered miRNA expression signature of irradiated PBL by decreasing the number of radio-responsive miRNAs. Integrated analyses of transcriptome and microRNAome suggested that modeled microgravity affected the DNA-damage response to irradiation [87]. Fu et al. analyzed RNA expression profiles in human lymphoblastoid TK6 cells incubated for 24 h under static or stimulated microgravity after 2 Gy γ-ray irradiation. Although no differentially-expressed miRNAs were identified under either simulated microgravity or irradiation conditions, both miR-15b and miR-221 were found to be differentially expressed under compound conditions with an interactive effect [88].

Furthermore, some studies were also carried out to gain insight into the effects of spaceflight on miRNA expression profile. Some researchers from Dr. Yeqing Sun’s group explored the miRNA expression profile changes in space-flown *C. elegans* larvae that experienced the 16.5-day shuttle spaceflight on Shenzhou-8 spacecraft. 23 miRNAs were found to express differentially in spaceflight groups compared with the ground control group and were predicted to be involved in the regulation of developmental processes, growth and body morphogenesis, DNA damage response as well as biological behavioural responses [89,90]. Gao et al. from the same team also investigated miRNAome and mRNA expression in the *ced-1 C. elegans* mutant vs. the wild-type strain, both of which underwent spaceflight, spaceflight 1g-centrifuge control and ground control conditions during the Shenzhou-8 mission, and found differential miRNA expression increased from 43 (ground control condition) to 57 and 91 in spaceflight and spaceflight control conditions, respectively, suggesting several miRNAs responding to space radiation were deregulated by microgravity, which is consistent with the finding of Girardi’s group. Some of differentially expressed miRNAs were predicted to regulate apoptosis, neurogenesis larval development and ATP metabolism [91].

Considering the above studies, we can see that for the same cell or tissue type, the miRNA expression profile is radiation type-, dose- and time-dependent. The miRNA expression profiles are also significantly different in varying cell or tissue types even though exposed to the same radiation of the same dose. This suggests that we should consider radiation type, dose, biological samples, as well as sample collection time comprehensively when designing space radiation-related miRNA experiments. In addition, we note that both miR-150-5p and miR-342-3p are down-regulated in blood samples of different mouse models (C57BL/6 or Kunming mice) at 24 h after exposed to different radiation (0.5 Gy iron or carbon ions) [80,82], implying their important regulatory roles in biological effects of heavy ion radiation. Although a large number of miRNAs have been identified to be involved in the responses to space radiation, few functional experiments were carried out. Zhu et al. found that high LET IR promoted miR-21 expression through the EGFR/STAT3 pathway and miR-21 played an important role in high LET IR-induced carcinogenesis [92,93]. Zhang et al. found miR-21 promoted formation of high LET (Fe ions) irradiation-induced reactive oxygen species (ROS) by targeting both SOD3 and TNFα and contributed to an elevation of IR-induced cell transformation [94]. Wang et al. found that radiation didn’t induce any lung tumorigenesis in miR-21^–/–^ mice while miR-21 knock-in mice showed high spontaneous lung tumor incidences. By further analysis, miR-21 was found to be upregulated by oxygen, silicon and iron ion irradiation and contribute to radiation-induced lung tumorigenesis, whereas its level in serum could be used as a biomarker for predicting high-LET radiation-induced lung tumorigenesis [95]. Kim et al. found high energy proton irradiation induced down-regulation of miR-31-5p in mouse serum while its inhibition protected human colonic epithelial cells against ionizing radiation in a hMLH1-dependent manner [96]. However, most studies involving space radiation-related miRNAs are limited to description of radiation-induced changes of miRNA profiles, the functions of the differentially expressed miRNAs and the underlying mechanisms in space radiation-induced biological effects are still not revealed.

## 4. Future Challenges

Increasing evidence shows that miRNAs function as significant molecules in the regulation of the response of living organisms to various kinds of space radiation. Based on the findings mentioned above, several specific miRNAs from varying kinds of exposed tissues have been associated with the space radiation response. Besides, it has been reported that serum miRNAs can be used as biomarkers in early stages after exposure to assess space radiation damage, and that five miRNAs (miR-183-5p, miR-9-3p, miR-200b-5, miR-342-3p, and miR-574-5p) present dose- and time-dependent changes upon different types of radiation exposure [85]. Thus, analyzing the expression profiling of miRNAs is expected to be conducive to assess risk of space radiation.

Space radiation cause expressional changes of different types of miRNAs, contributing to the change of the corresponding mRNAs, related genes and other regulatory factors, which pose a significant impact on various biological processes of an organism. Therefore, exploring the biological functions and the underlying action mechanisms of the differentially expressed miRNA is of great significance to the prediction of the medical results caused by space radiation. However, the biological importance of the miRNAs responding to space radiation is incompletely understood. In fact, most of the currently reported space radiation-related miRNA research is limited to the description of the expressional patterns of miRNAs (up- or down-regulation). The exact contribution of the differentially expressed miRNAs to the specific health effects of the space radiation is required to be deciphered for applying them for space radiation risk prediction and possible therapy of the lesions caused by space radiation, which depends on the target identification and tissue-specific functional analysis. Besides the focus on miRNA profiling in response of the organisms to space radiation, more attention should be paid to the profiling of mRNAs and proteins responding to space radiation at the same time since miRNAs play regulatory roles through translation inhibition or mRNA degradation of the target genes. Further, recent studies have shown miRNA is involved in competing endogenous RNA (ceRNA) regulatory network with long non-coding RNA (lncRNA) or circular RNA (circRNA). Taking these issues into account, new approaches in genomics, transcriptomics, proteomics, metabolomics and epigenomics should also be employed in the mechanistic study, which will help to elucidate the effects of space radiation and to develop personalized countermeasures more precisely.

Due to the nature and characteristics of the space radiation, space radiation-related miRNA studies should be improved in the following two aspects. First, more attention should be paid to the miRNAs involved in the space radiation-induce bystander effect. Considering the low dose/dose rate and low fluence characteristics of space radiation, most cells of the exposed living organisms will not be hit by the space high energy particles, thus the radiation-induce bystander effect plays a significant role in the space radiation-induced biological effects in this situation [97]. It has been found that several miRNAs play important roles in radiation-induced bystander effects [98,99], however, there is no reports about miRNAs functioning in the space radiation-induced bystander effects so far. Second, more studies should be performed to identify miRNAs participating in the regulation of compound effect of space radiation and other space environmental factors, such as microgravity, weak magnetic field, and short circadian rhythm. The interactive effects between the mixed radiation as well as space radiation and other space environmental factors have been observed in a number of studies [100,101,102,103]. A few researchers probed into the miRNA profiling changes in response to ionizing radiation and microgravity [88], while there is still lack of studies with all factor simulation of space environment, which is dependent on the building up of ground-based all factor space radiation simulation platform. As for the miRNA study relating to mixed space radiation, there are no reports so far. In conclusion, space radiation-related miRNA study has made significant achievements and is showing tremendous research values. However, there still is a lot of work to do for space radiobiologists all around the world before a better understanding of both functions and mechanisms of the space radiation-responding miRNAs could be obtained as well as these space radiation-responding miRNAs could be used as *bona fide* biomarkers for space radiation risk assessment.

## Figures and Tables

**Table 1:** Studies relating to miRNAs responding to space radiation.

Studied Materials	Radiation	MiRNA Expression	Target Proteins/Pathways	MiRNA Detection Methods	Predicted Biological Consequences	Refs
Testis, brain and liver tissues of Balb/C male mice, 4 h after exposure	2 Gy whole-body proton irradiation	Dysregulation of 14 mouse testis, 8 liver, and 8 brain miRNAs, with 20 up- and 10 down-regulated	-	NGS for profiling, no validation	Regulation of DNA damage response and tumorigenesis	[78]
Blood of C57BL/6 male mice, 6 h/24 h after exposure	0.5 or 1.0 Gy whole-body proton irradiation	Dysregulation of 26 miRNAs, with 5 up- and 21 down-regulated	-	qRT-PCR	Regulation of nucleic acid metabolic process and system development	[79]
Bystander 3-D artificial human tissues, 8 h, 1, 2, 3, 4, 5 and 7 days post-IR	5.4 Gy α particle irradiation for the first layer of cells	Dysregulation of 12 miRNAs, with 7 up- and 5 down-regulated	DNMT3a, MCL1, BCL2, RB1, E2F1/ apoptosis pathway, TNF signaling pathway, cellular senescence pathway	miRNA microarray for profiling, no validation	Apoptosis, cell cycle deregulation and DNA hypomethylation	[80]
Three human cell lines (A549, THP-1 and HFL), 24 h/72 h after exposure	0.5, 1.0 or 1.5 Gy α particle irradiation	Dysregulation of 13 miRNAs, with 8 up- and 5 down-regulated	-	miRNA microarray for profiling, qRT-PCR for validation	Regulation of ribosomal assembly, lung carcinoma development, TGF-β signaling, cell communication, etc	[81]
Immortalized human bronchial epithelial cells (BEAS-2B), 40 passages after exposure	0.1, 0.25, 0.5 or 1 Gy α particle irradiation	Dysregulation of 68 miRNAs, with 20 up- and 48 down-regulated	PEG10, ARHGAP26, IRS1/β-catenin pathway, PI3K-Akt signaling pathway, TGF-β signaling pathway	miRNA microarray for profiling, qRT-PCR for validation	Promotion of malignant transformation	[82]
Blood of C57BL/6 male mice, 6 h/24 h after exposure	Whole-body irradiation by 0.1 or 0.5 Gy iron ions	Dysregulation of 14 miRNAs, with 6 up- and 8 down-regulated	-	qRT-PCR	Regulation of transcription, nucleic-acid metabolism, and development	[83]
Testis of Swiss-Webster male mice, 4 weeks after exposure	Whole-body irradiation by 2 Gy carbon ions	Dysregulation of 70 miRNAs, with 56 up- and 14 down-regulated	-	NGS for profiling, qRT-PCR for validation	Apoptosis of spermatogenic cells	[84]
Blood of Kunming male mice, 24 h after exposure	Whole-body irradiation by 0.5 or 2 Gy carbon ions	Dysregulation of 12 miRNAs, with 6 up- and 6 down-regulated	-	miRNA microarray for profiling, qRT-PCR for validation	Regulation of cell cycle transition, immune system and carcinogenesis	[85]
Blood of Kunming male mice, 24 h after exposure	Whole-body irradiation by 0.25 or 0.5 Gy carbon ions	Upregulation of let-7a-5p and miR-200b-5p	-	miRNA microarray for profiling, qRT-PCR for validation	-	[86]

**Table 2:** Studies relating to miRNAs responding to space compound environment.

Studied Materials	Experimental Treatments	MiRNA Expression	Target Proteins/Pathways	MiRNA Detection Methods	Predicted Biological Consequences	Refs
Human peripheral blood lymphocytes	4 or 24 h incubation in modeled microgravity after irradiation with 0.2 or 2 Gy γ-rays	Downregulation of let-7i*, miR-7, miR-7-1*, miR-144, miR-200a, miR-598, miR-650, upregulation of miR-27a, miR-99b by combined action	ATM, FANCF, STAT5A, BAX/DNA damage response pathway, p53 pathway	miRNA microarray for profiling, qRT-PCR for validation	Modeled microgravity inhibits the DNA-damage response to IR	[87]
Human lymphoblastoid TK6 cells	24 h incubation under simulated microgravity after irradiation with 2 Gy γ-rays	Hsa-miR-15b was downregulated while hsa-miR-221 was upregulated by combined action	Apoptosis pathway, NF-κB pathway, TNF signaling pathway	miRNA microarray for profiling, qRT-PCR for validation	Regulation of apoptosis process and immune response by combined action	[88]
*C. elegans* (Bristol N2)	7 h after a 16.5-day shuttle spaceflight on Shenzhou-8, with 1.92 mGy space radiation in static slot and 2.27 mGy in centrifuge slot	The expression of 23 miRNAs changed when exposed to a space synthetic environment and a space radiation environment	Capg-2, deb-1, ZK180.5, egl-5, C07H4.1, wrk-1, nep-11, odr-2, eff-1, air-2, bath-41, etc/DNA damage response pathway, apoptosis pathway	miRNA microarray for profiling, qRT-PCR for validation	Regulation of embryonic development, growth and body morphogenesis, biological behavioural responses, DNA damage response, etc	[89,90]
*C. elegans* (wild-type strain vs. *ced-1* mutant)	7 h after a 16.5-day shuttle spaceflight on Shenzhou-8, with 1.92 mGy space radiation in static slot and 2.27 mGy in centrifuge slot	Differential miRNA expression increased from 43 (ground control condition) to 57 and 91 in spaceflight and spaceflight control conditions	Ced-10, drp-1, hsp-1/ DNA damage response pathway, p53 pathway	miRNA microarray for profiling, qRT-PCR for validation	Regulation of apoptosis, neurogenesis larval development, ATP metabolism and GTPase-mediated signal transduction	[91]

The asterisk (*) indicates a miRNA expressed at low levels relative to the miRNA from the opposite arm of the same pre-miRNA hairpin.
